# Covalent Inhibition of the *Human* 20S Proteasome with Homobelactosin C Inquired by QM/MM Studies

**DOI:** 10.3390/ph15050531

**Published:** 2022-04-25

**Authors:** Natalia Serrano-Aparicio, Silvia Ferrer, Katarzyna Świderek

**Affiliations:** BioComp Group, Department of Physical and Analytical Chemistry, Universitat Jaume I, 12071 Castellón de la Plana, Spain; serranon@uji.es (N.S.-A.); sferrer@uji.es (S.F.)

**Keywords:** 20S proteasome, covalent inhibition, β-lactone, homobelactosin C, QM/MM, molecular dynamic, molecular mechanism, free energy, umbrella sampling, M06-2X

## Abstract

20S proteasome is a main player in the protein degradation pathway in the cytosol, thus intervening in multiple pivotal cellular processes. Over the years the proteasome has emerged as a crucial target for the treatment of many diseases such as neurodegenerative diseases, cancer, autoimmune diseases, developmental disorders, cystic fibrosis, diabetes, cardiac diseases, atherosclerosis, and aging. In this work, the mechanism of proteasome covalent inhibition with bisbenzyl-protected homobelactosin C (hBelC) was explored using quantum mechanics/molecular mechanics (QM/MM) methods. Molecular dynamic simulations were used to describe key interactions established between the hBelC and its unique binding mode in the primed site of the β5 subunit. The free energy surfaces were computed to characterize the kinetics and thermodynamics of the inhibition process. This study revealed that although the final inhibition product for hBelC is formed according to the same molecular mechanism as one described for hSalA, the free energy profile of the reaction pathway differs significantly from the one previously reported for γ-lactam-β-lactone containing inhibitors in terms of the height of the activation barrier as well as the stabilization of the final product. Moreover, it was proved that high stabilization of the covalent adduct formed between β5-subunit and hBelC, together with the presence of aminocarbonyl side chain in the structure of the inhibitor which prevents the hydrolysis of the ester bond from taking place, determines its irreversible character.

## 1. Introduction

The ubiquitin-proteasome system (UPS) is responsible for hydrolyzing more than 90% of all cytosolic proteins, where the 26S proteasome, a large ~2.5 MDa molecular complex, is the central player in this non-lysosomal protein degradation pathway [1]. The 26S proteasome is formed by two subcomplexes, the catalytic core particle (CP) or 20S proteasome, and one or more 19S regulatory particles (RP) that serve as activators. This complex system was discovered in 1969 when an ATP-dependent proteolysis process was identified [2]. Later it was connected to a stable polypeptide, named ubiquitin for being present in all tissues of eukaryotic organisms [3,4]. Eventually, the intracellular proteolytic system was identified as a big multimolecular protein initially called “cylindrin” due to its cylindric shape [5], afterward named as the 20S proteasome [6,7,8] and confirmed to be the catalytic core of the ATP dependent proteolysis in the UPS [9]. An overall achievement in the characterization of the ubiquitin conjugation system and its role in specific proteolysis labeling was awarded with the Nobel Prize in Chemistry in 2004 to Avram Hershko, Aaron Ciechanover, and Irwin Rose [10].

It was discovered that the UPS degrades proteins in a multistep process, where target proteins are linked by covalent bonds to the ubiquitin in presence of ATP [11,12]. This small protein binds to the 19S RP of the 26S proteasome, where the protein destined for degradation is unfolded and inserted into the multicatalytic chamber, the CP, to be rapidly discomposed into smaller polypeptides [13,14,15]. Since the 20S proteasome’s primary role is to degrade key regulatory and aberrant proteins [16], it procures the maintenance of cellular homeostasis controlling protein turnover. [15] Being the main protein degradation pathway in the cytosol, UPS has a wide spectrum of polypeptide substrates, and its function intervenes in multiple crucial cellular processes [17]. The UPS participates in cell cycle progression, [18,19,20] cellular signaling [21,22], genome integrity [23,24], regulation of endocrine pathways [25,26], apoptosis [27,28], transcriptional regulation [29,30], metabolism regulation [31,32], immune responses [33,34], and in various human diseases and pathogenesis [35,36]. Therefore, all these functions make the proteasome a crucial target for therapeutic intervention against, for instance, neurodegenerative diseases [37,38], cancer [39,40,41], autoimmune diseases, developmental disorders, cystic fibrosis, diabetes, cardiac diseases, atherosclerosis, or just aging [42]. Some infectious diseases such as Chaga’s disease [43], malaria [44,45,46], and tuberculosis [47] have been also related to proteasomal function.

**Figure 1:**
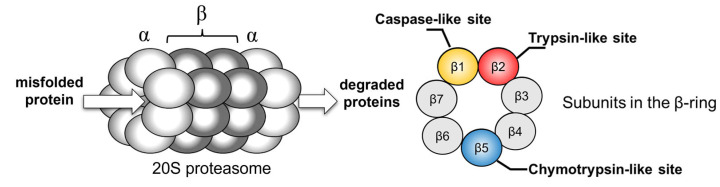
Schematic representation of constitutive 20S proteasome with the indicated position of catalytically active subunits in β-rings.

Except for bacteria (excluding actinomycetes), which harbor complexes of two homohexameric rings with one active subunit each [48], the 20S proteasomes consist of four stacked homoheptameric rings formed by two types of basic subunits known as α and β in the rest of living organisms. As shown in Figure 1 each of the two outer rings is formed by seven α subunits and each of the two inner rings by seven β subunits.

The α proteins have a key role in the complex assembly due to the formation of the entry gate to the interior of the proteasome. The β subunits bear the catalytic activity. [49,50] In eukaryotes, only three active β subunits (i.e., β1, β2, and β5 in constitutive variant) were found, that present different specificity towards the substrates due to the different architecture of S1 pockets located in their binding sites. The β5 subunit, characterized by the presence of Met45 in the hydrophobic S1 pocket, is known as chymotrypsin-like protease. The β2 subunit with hydrophilic S1 cavity with negatively charged Asp55 residue is known as trypsin-like protein. Finally, the β1 owes its caspase-like character to positively charged Arg45 residue located in the bottom of its hydrophilic pocket [51]. 

Most known proteasome inhibitors are peptide-like molecules bearing electrophilic warheads that reversibly or irreversibly bind to the catalytic O^γ^Thr1 forming covalent adducts. Therefore, proteasome inhibitors are commonly classified according to the type of functional groups of this reactive portion of the molecule. Seven different inhibitor classes are recognized at the moment, i.e., aldehydes, boronic acids, α,β-epoxyketones, α-ketoaldehydes (glyoxals), vinyl sulfones, vinyl amides (syrbactins), and β-lactones [52]. So far, only two boronic-acid, Bortezomib (Velcade®, Millennium Pharmaceuticals, Cambridge, MA, USA), and Ixazomib (Ninlaro^®^, Takeda Pharmaceuticals, MA, USA), and one α,β-epoxyketone, Carfilzomib (Kyprolis®, Amgen Inc., Thousand Oaks, CA, USA), have been approved by the U.S. FDA [53,54,55,56,57]. All three molecules inhibit the β5/β5i subunit. 

**Figure 2:**
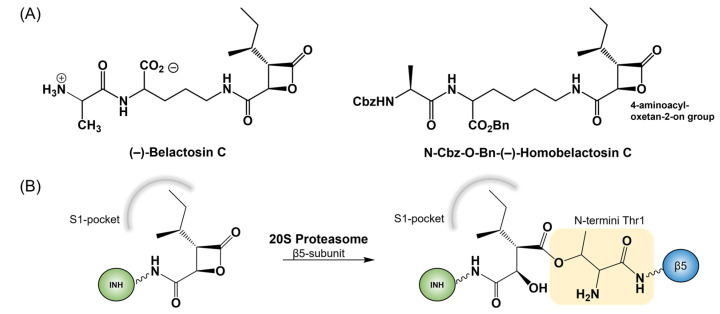
Structure of (−)-Belactosin C and bisbenzyl-protected N-Cbz-O-Bn-(−)-Homobelactosin C (**A**) (where Bn stands for a benzyl group and Cbz for benzyl carbamates) together with the general scheme for the covalent inhibition reaction (**B**).

Herein, the inhibition mechanism of the β5 subunit from *human* 20S proteasome with the homo-analog of belactosin C (BelC) was explored using a computational approach including molecular dynamic (MD) simulations with classical and quantum mechanics/molecular mechanics (QM/MM) potentials. Originally, isolated from *Streptomyces* sp. UCK14, the BelC was found to exhibit antitumor activity [58,59]. This activity was shown to increase significantly upon acetylation of the free amino group and esterification or amidation of the carboxyl group, as well as displacement of the ornithine moiety with lysine, resulting in the homo-analog homobelactosin C. Both structures of BelC and its homolog are shown in Figure 2A. Introduced modifications to the structure of BelC resulted in IC50 on the nanomolar level measured against human pancreoma and colon cancer [60,61]. This high antitumor activity was related to proteasome inhibition [62,63,64,65]. The crystal structure obtained for *yeast* CP [66] in a complex with bis-benzyl-protected homobelactosin C (hBelC) covalently bound in the active site suggested that the β-lactone (equally as other covalent proteasome inhibitors) undergoes nucleophilic attack by oxygen gamma of N-termini threonine (O^γ^Thr1) resulting in ester bond formation, as shown in Figure 2B. Nevertheless, it was demonstrated in the same study that this inhibitor presents high selectivity towards the primed site of the chymotrypsin-like β5 active site, adopting a completely different orientation to the one observed previously for β-lactone-containing proteasome inhibitors such as omuralide (Omu) or salinosporamide A (SalA). Moreover, based on the final product orientation, it was suggested that hBelC follows a unique mechanism preventing cleavage of its covalent adduct formed with the active site. The resistance of the ester bond was assigned to the presence of a 4-aminocarbonyl side chain that according to crystallographic studies can block the access of the water molecule due to its specific orientation in the binding pocket. 

Therefore, the main goal of this work was to clarify the mechanism of inhibition involving hBelC, including recognition and inactivation steps. Insight into the specific interactions established between the inhibitor and residues from the primed site of the enzyme can be very helpful for several reasons. Mainly, it can provide a guideline that can be used to refine the recognition step by eliminating unfavorable interactions and improving the existing favorable ones. This is especially important in new strategies used to design polypharmacological anticancer drugs [67] where hBelC could serve as a template for the dual inhibition of the proteasome and fatty acid as proposed by Romo and co-workers. [61] Moreover, the information gathered at the molecular level on the ligand interaction with this part of the active site may contribute to a better understanding of the catalytic activity of the β5 subunit, especially since little is known about interactions of the substrate with this region. 

In the case of the inactivation step, the mechanism of inhibition was studied, and the structure of the final product complex was analyzed to shed light on the hydrolysis step of the newly formed covalent adduct. Free energy surfaces were explored assuming the most favorable molecular mechanism, which was proposed based on our previous experience. Finally, a comparative analysis of results obtained for the hBelC and γ-lactam-β-lactone, homo-salinosporamide A (hSalA) was carried out to provide information on similarities and differences in the inhibition process offered by molecules belonging to the same warhead family.

## 2. Results and Discussion

In general, covalent inhibition is a two-step process. [68,69] First, an inhibitor reversibly associates with the target enzyme, and a protein-inhibitor complex (*E*-*I*) is formed. The potency of this first step is defined by the binding constant *K_i_*. In the second step, the reaction occurs between the two reactive entities of the inhibitor and the enzyme, respectively, forming a protein-inhibitor covalent complex (*E*-*I*). This chemical transformation is characterized by the rate constant of inactivation, *k_inact_* as summarized in Equation (1)

(1) $$E+I\overset{K_{i}}{↔}E•I\overset{k_{inact}}{\to }E-I$$

where *E* stands for the enzyme, *I* is an inhibitor, *E*•*I* is a non-covalent enzyme-inhibitor complex, and *E*-*I* represents a covalent adduct formed between enzyme and inhibitor. Computational research on covalent inhibitors often focuses solely on the energetics of the kinetic step, as high accuracy insights into the binding process seem to be still beyond the scope of currently available in silico techniques. Nevertheless, in this work, long MD simulations with a classical force field were carried out for the covalent E-hBelC complex to shed light on the recognition step. Subsequently, the mechanism of covalent adduct formation that occurs in the inactivation process was explored using QM/MM methods. The obtained results for both steps are described below. 

### 2.1. Recognition Step

A key part of the optimization of inhibitors in pharmaceutical drug development is to vary the molecular design to enhance the complementarity of the chemical features of the compound with the position of the side chains of the amino acids in the binding pocket of a target enzyme [70]. In the special case of the covalent inhibitors, the recognition step requires an additional condition to be fulfilled. This is related to the optimal relative position of the warhead with respect to the catalytically important residues to facilitate the covalent adduct formation. According to crystallographic data, hBelC seems to fulfill both requirements satisfactorily. Nevertheless, because MD simulations were carried out in the final product of inhibition, the position of the warhead will not be analyzed here in detail, and therefore all attention will be focused on the behavior of the inhibitor tail within the binding cavity.

**Figure 3:**
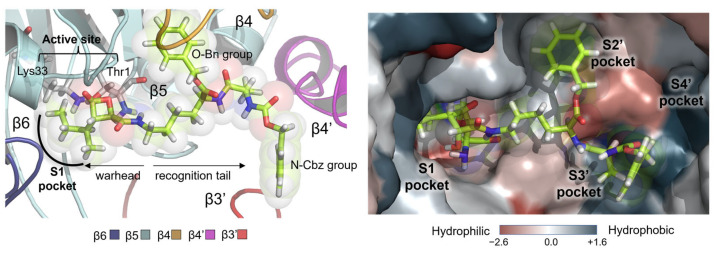
Structure of the binding site of N-Cbz-O-Bn-(−)-homobelactosin C in *human* 20S Proteasome with indicated positions of the Thr1 and Lys33, catalytically important residues in the active site and five key subunits β6, β5, β4, β3′and β4′ that participate in its formation (on the (**left**)) together with its hydrophobicity map generated based on the Eisenberg hydrophobicity scale (on the (**right**)).

The shape of the site that binds hBelC to the active site of the β5 chain is, in fact, determined by the orientation of five subunits contained in the two β rings of the proteasome, i.e., β6, β5, and β4 subunits from one ring, and β3′ and β4′ from the neighboring one. As shown in Figure 3, the biggest portion of the hBelC inhibitor is bound to the β5 subunit that involves an active site together with the S1 pocket. Analysis of the hydrophobicity map confirms the existence of well-known high hydrophobicity of this pocket, at the same time indicating the hydrophilic character of the active site due to the presence of Lys33 with the hydrophobicity of −1.5 according to Eisenberg hydrophobicity scale [71] and Thr1 with the hydrophobicity of −0.05. Due to the molecular characterization of the inhibitor structure, the S1′ pocket remains empty and consecutive S2′ and S3′ pockets of the primed site are occupied by O-Bn (benzyl group) and methyl group, respectively. 

**Figure 4:**
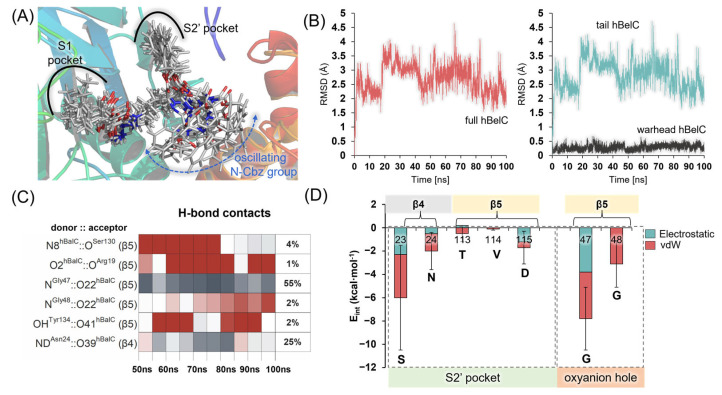
(**A**) Eleven snapshots illustrating a variety of conformations explored by hBelC in the binding cavity of 20S proteasome during the last 50 ns of MD simulations. (**B**) The root-mean-square-deviation computed for positions of heavy atoms of hBelC in its covalent complex with 20S proteasome during 100 ns of classical MD simulations**.** (**C**) H-bond contacts established between hBelC and residues from the binding pocket during the last 50 ns of simulations defined using geometric criteria (3.0 Å for Donor···Acceptor distance, and 135.0° for Donor-H⋯Acceptor angle) (**D**) Interaction energies computed for hBelC and residues of S2′ pocket as well as residues of the oxyanion hole.

It was observed that the N-Cbz group (benzyl carbamate) attached at the end of the hBelC tail does not enter the S4′ pocket, even though this pocket is highly hydrophobic. The hydrophobic character of this pocket is achieved due to the presence of Ile25 and Val26 of the β4, Val32 of the β5, and Phe170 and Ile171 of the β4′ subunit with the values of the hydrophobicity ranging between +1.08 and +1.38 (as provided by the Eisenberg scale). It is possible that the behavior of this N-Cbz group originates in its large size or is dictated by the attachment of the benzyl group to the sp^2^ oxygen instead sp^3^ carbon atom, as would be expected for the side chain of polypeptide substrate. In consequence, the presence of an oxygen atom in this position may force the aromatic ring to adopt an alternative orientation from desired in the cavity. The lack of geometrical complementarity of the N-Cbz group towards this pocket results in its high mobility as shown in Figure 4A,B where the various conformations explored by the N-Cbz group within the active site are shown together with the evolution of root-mean-square-deviation (RMSD) computed for the heavy atoms of the inhibitor during the simulations. As shown on the plots, the inhibitor experiences the highest deviations from the original position due to the flexibility of its tail. This finding proves that the N-Cbz group does not create specific interactions within the binding site of the 20S proteasome and is not crucial for the recognition step. 

On the other hand, the O-Bn group seems to play an important role, because it binds very tightly inside the corresponding S2′ pocket and remains there during the overall MD simulations. The size and shape of this pocket seem to be established by the presence of the side chains of three residues located on its bottom, Asp115, Val114, and Tyr113, by the beta-sheet, that consists of beta-strands of the β5 subunit on one side, and by the backbone of Ser23, and the side chain of Asn24 from the β4 subunit on the other. Favorable interactions, as shown in Figure 4, created between these residues and hBelC ensure the stability of its binding. 

Finally, the hydrogen bond (H-bond) contacts established between hBelC and the binding cavity during MD simulations were determined (see Figure 4). Based on the obtained results, we concluded that it is rather unlikely that the recognition step depends on this type of interaction. In addition to Gly47, which is involved in H-bond interaction with the carbonyl oxygen of the β-lactone ring, only one more H-bond was identified, established between the NH_2_-group of the side chain of Asn24 from the β4 chain and the oxygen of the peptide bond from the backbone of the side-chain of lactone ring of the hBelC. In all, the primed site occupied by this inhibitor was not found to provide more H-bond interactions that could be indicated as key for the recognition step. 

It is very likely that the minimum requirement for an inhibitor structure to form a non-covalent adduct with the active site of the β5 subunit of the *human* 20S proteasome is primarily related to the molecular architecture of the P1, as has been proven for Omu or SalA. However, it seems that in the case of hBelC where a small P1 group is employed, the presence of the O-Bn group in the P2′ position may play an important supporting role.

### 2.2. Inactivation Step

In our previous studies, the mechanisms of inhibition with promising candidates, such as dihydroeponemycin [72] and SalA [73], i.e., α,β-epoxyketone, and γ-lactam-β-lactone, have been revealed using QM/MM MD methods. Based on computationally delivered results supported by the newest experimental data [74], it was concluded that the originally proposed and widely accepted mechanism of inhibition suggesting that only one residue of the active site, N-termini Thr1 [75], is involved in the process was incomplete. As demonstrated by obtained results, active participation of another residue, i.e., Lys33 was required to accomplish inhibition within a realistic energetic cost. [72] The role of this residue in the inactivation step of inhibition was further confirmed in studies focused on homo-salinosporamide A (hSalA) [76].

**Figure 5:**
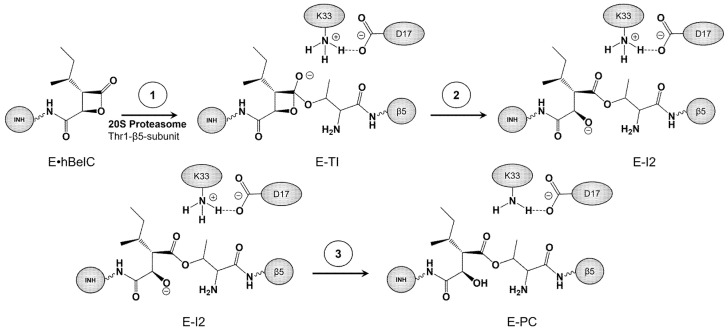
Schematic representation of the proposed three-step inhibition mechanism of β5-subunits of the 20S proteasome with N-Cbz-O-Bn-(−)-homobelactosin C.

Therefore, guided by the previously acquired knowledge, we assume that in the case of the hBelC, the inhibition mechanism should be very close to the one previously described for hSalA. Thus, it was proposed, that the inhibition process takes place in three steps, as illustrated in Figure 5.

Accordingly, it was proposed that in the first step, oxygen gamma (O^γ^) of Thr1 is activated by nitrogen zeta (N^ζ^) of Lys33, which abstracts the hydrogen gamma (H^γ^), and consequently, the O^γ^ atom attacks the carbonyl carbon (C1^βlac^) of the β-lactone ring generating a covalent adduct in the form of a tetrahedral intermediate (E-TI). In the second step, the bond between carbonyl carbon (C1^βlac^) and the oxygen (O2^βlac^) of β-lactone is cleft resulting in a ring-opening. This step is accompanied by the accumulation of negative charge on O2^βlac^. As demonstrated in our previous work, [76] such a newly formed adduct is expected to be thermodynamically unfavorable, and thus an additional chemical step was proposed to neutralize the separation of charges observed in the active site of intermediate 2 (E-I2), i.e., the negative charge of the oxygen (O2^βlac^) of the open lactone ring and positive charge on nitrogen zeta (N^ζ^) of Lys33. A desired final neutral product may be obtained, as proposed previously, [76] by rearranging the positions of hydrogen atoms within the active site. This rearrangement could be achieved by a double proton transfer that would involve the transfer of one proton from Lys33 to the nitrogen (N^Thr1^) of the amino group of Thr1 and the second proton from the same amino group to the oxygen (O2^βlac^) of the open β-lactone. Formation of the stable product of inhibition (E-PC) is expected once this last process is accomplished.

**Figure 6:**
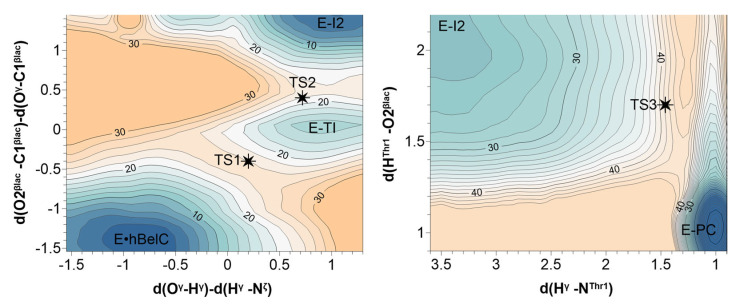
Free energy surfaces explored for all three proposed steps of the chemical transformations occurring along the 20S proteasome inhibition mechanism with N-Cbz-O-Bn-(−)-homobelactosin C. PMFs were obtained at AM1/AMBER level and corrected at M06-2X/AMBER level of theory with 6-31+G(d,p) basis set (see Computational Methods for details). Values of distinguished coordinates and energy are given in Å and in kcal·mol^−1^, respectively. Black stars indicate the positions of optimized and characterized transition state structures detected along the reaction progress at the M06-2X/AMBER level of theory with a 6-31+G(d,p) basis set.

The existence of E-TI was previously excluded based on experimental studies on β-lactone inhibitors in serine proteases [77] in which it was suggested that the tetrahedral transition state may not be generated in the acylation reaction pathway, due to the high ring strain energy within the β-lactone. Consequently, we decided to explore the evolution of the first and second steps on the same free energy surface to provide evidence on whether the nucleophilic attack and ring-opening take place in a stepwise manner. Therefore, both chemical transformations were explored by controlling the antisymmetric combination of the d(O^γ^-H^γ^) and d(H^γ^-N^ζ^) distances, which describe the process of proton transfer from Thr1 to Lys33, together with the antisymmetric combination of d(O2^βlac^-C1^βlac^) and d(O^γ^-C1^βlac^) distances that define the process of β-lactone ring-opening and nucleophilic attack of Thr1 on its carbonyl carbon, respectively. The computed free energy surface is provided in Figure 6. 

**Figure 7:**
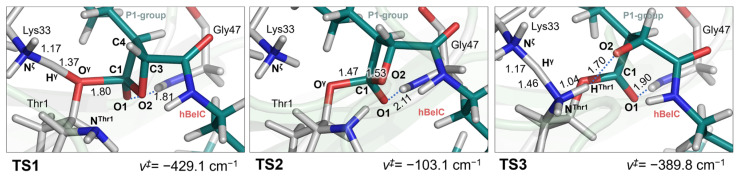
Optimized and characterized transition state structures along the minimum energy reaction pathway at the M06-2X/AMBER level of theory with a 6-31+(d,p) basis set. Key distances are in Å while values of imaginary wavenumbers are in cm^−1^.

The minimum free energy pathway from non-covalent adduct (E-hBelC) leads, as originally assumed, to a metastable tetrahedral intermediate (E-TI) through the first transition state (TS1). Subsequently, the system is crossing the second transition state (TS2) to form the second intermediate (E-I2). The existence of both, TS1 and TS2 was confirmed by optimization and characterization of their structures at the M06-2X/AMBER level of theory as shown in Figure 7. All key distances and charges obtained for optimized stationary structures and determined during reaction progress are provided in Tables S1 and S2 of Supplementary Materials.

Subsequently, the free energy surface for the last step was explored controlling the d(H^γ^-N^Thr1^) distance describing the transfer of the proton (H^γ^) originally located on Lys33 to the nitrogen atom (N^Thr1^) of NH_2_-group of Thr1, together with the d(H^Thr1^-O2^βlac^) distance that describes second proton transfer from the amino group of threonine to the negatively charged oxygen of open β-lactone ring (see Figure 6). The minimum energy path indicates that both protons are transferred in a concerted way, and the NH_2_- group of Thr1 serves as a proton shuttle in the inactivation step. A detail of the structure of the transition state (TS3) for the last step of the inhibition mechanism, optimized at the M06-2X/AMBER level of theory, is shown in Figure 7. 

**Figure 8:**
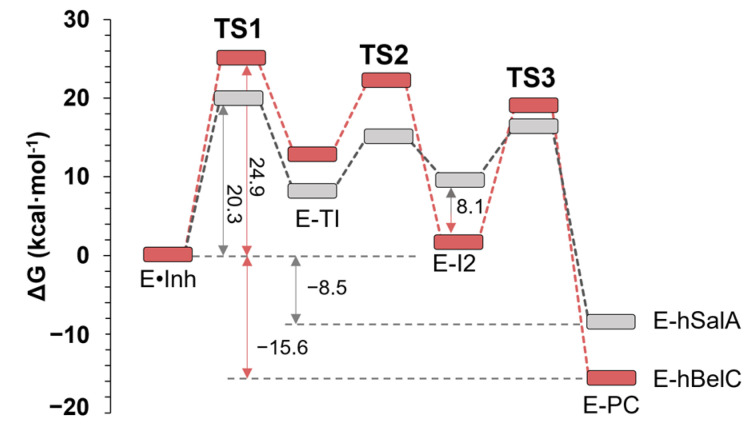
Free energy profile computed for 20S proteasome inhibition mechanism by N-Cbz-O-Bn-(−)-homobelactosin C (hBelC) (in red) and homo-Salinosporamide A (hSalA) (in gray). Results for hSalA were taken from [76].

Both computed free energy surfaces were consequently used to obtain the free energy profile for the complete inactivation step of the inhibition which is presented in Figure 8. Since the mechanism of inhibition with hBelC and hSalA appear to be identical considering the sequence of events occurring during the chemical reaction, the newly obtained energetic profile was further confronted with the same profile computed for the inhibition of β5-subunit of the 20S proteasome with hSalA, deduced from our previous work [76].

**Figure 9:**
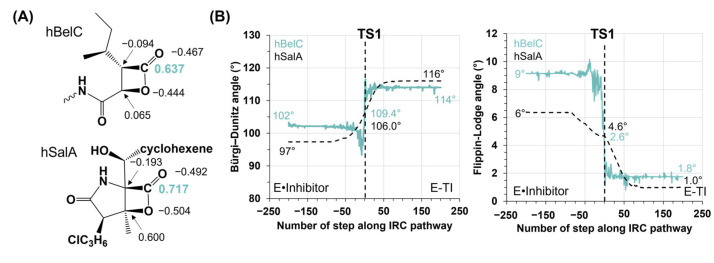
(**A**) Distribution of ESP charges on the β-lactone ring in hBelC and hSalA inhibitors computed using ChelpG method at M06-2X/6-31G+(d,p) level of theory with 6-31+G(d,p) basis set in gas phase. (**B**) Evolution of nucleophilic attack trajectory described by Bürgi–Dunitz (α_BD_) and Flippin–Lodge (α_FL_) angles along minimum energy pathway computed at M06-2X/6-31G+(d,p) level of theory for the first step of 20S proteasome inhibition with hBelC and hSalA.

According to the computed Gibbs free energy profile, it was found that the rate-determining step corresponds, as in other cases of β-lactone containing inhibitors, to the first chemical step, i.e., the nucleophilic attack of Thr1 on the β-lactone ring. However, and surprisingly, the obtained free energy barrier of 24.9 kcal·mol^−1^ for hBelC is meaningfully higher than the one computed for molecules with a γ-lactam-β-lactone warhead that were ranging between 20.3 and 20.4 kcal·mol^−1^ for SalA and hSala, respectively [73,76] This different result for hBelC may be caused by the higher flexibility of the single β-lactone ring due to (i) the presence of the sec-butyl group in the P1 position, and (ii) the absence of γ-lactam ring in the warhead structure that induces rigidness of the ring and ensures the position of the electrophilic center towards the oxygen gamma of Thr1. The sec-butyl can be responsible for weaker recognition characteristics than the hydroxymethyl-cyclohexenyl group of SalA or hSalA. Alternatively, the high barrier could simply originate in the weaker electrophilic center offered by the smaller warhead of hBelC. To explore these possible scenarios, first, the ESP partial charges were computed on each atom belonging to the warheads of hBelC and hSalA inhibitors in gas phase. As shown in Figure 9A, the carbonyl carbon (C1^βlac^) of the β-lactone ring carries a higher positive charge in the case of hSalA (0.717 e^−^) than in hBelC (0.637 e^−^) due to its intramolecular characteristic. Therefore, a lower positive charge computed on C1^βlac^ in hBelC confirms that this warhead should provide a weaker electrophilic center. Although a relatively small shift of 0.08 e^−^ between charge distribution on C1^βlac^ atom in the β-lactone with and without attachment of a γ-lactam ring was observed, nevertheless it can result in meaningful change in the free energy barrier. In fact, such an effect was already observed previously in the case of C–N bond hydrolysis catalyzed by *Candida antarctica* lipase B where the charge shift of only 0.12 e^−^ on the nucleophile acceptor determined the possibility of the reaction taking place [78].

Possible deviation of the β-lactone ring of hBelC from the optimal position was examined by analyzing the electrophile-nucleophile distance together with the Bürgi–Dunitz [79,80,81] (α_BD_) and Flippin–Lodge [82] (α_FL_) angles that describe the nucleophilic attack trajectory. The obtained values of α_BD_ and α_FL_ were compared with those determined for the first step of inhibition with hSalA. The evolution of these two angles along the progress of the first step collected for hBelC and hSalA inhibitors is shown in Figure 9B As can be seen, the relative position between the nucleophile (O^γThr1^) and electrophilic center (C1^βlac^) slightly changes when comparing both Michaelis complexes. It was found that while in E•hBelC complex the α_BD_ of 102° is closer to the value of 105 ± 5° (value determined by Bürgi et al. as the angle that ensures a reliable position for the nucleophile to attack) than in E•hSalA complex (α_BD_ = 97°), and the estimated value of α_FL_, that in the ideal case is expected to be 0°, presents higher deviation of the nucleophile from the normal plane to the electrophile plane in E•hBelC complex (α_FL_ = 9°) than in E•hSalA (α_FL_ = 6°). Thus, a higher deviation of α_FL_ angle from the ideal angle equal to 0° together with the lower divergence of α_BD_ versus the ideal angle of 105°, measured at the initiation point of the first step of inhibition, do not allow for the conclusion that the increment of the free energy barrier computed for the first step of inhibition with hBelC is related to the energetic cost of the nucleophile and electrophile position rearrangement. Moreover, it is rather expected that due to the opposite behavior of α_BD_ and α_FL_ angles their overall contribution to energetic change would rather cancel out and the energetic barrier should be closer to the one computed originally for hSalA.

On the contrary, the electrophile-nucleophile distance was found to be longer in E•hBelC complex (2.46 Å) than in E•hSalA (2.35 Å). Therefore, elongation of the electrophile-nucleophile distance together with a weaker electrophilic center offered by hBelC can account as energetically unfavorable factors that can explain the rise in the barrier height observed when the hBelC is used to block the 20S proteasome activity.

The barrier of the rate-limiting step is not the only difference found when comparing the energetic profiles computed for the inhibition process with hBelC and hSalA. As shown in Figure 8, meaningful dissimilarity in the stabilization of intermediate 2 (E-I2) was detected. This complex, as described before, is a covalent adduct of Thr1 with an open β-lactone ring whose creation is accompanied by N^ζLys^(+) and O2^βlac^(−) ionic pair formation. Interestingly, while in hSalA this complex is c.a. 10 kcal·mol^−1^ higher than the non-covalent complex (E•hSalA), in hBelC it is almost equally stable as E•hBelC, with computationally predicted relative free energy of 1.9 kcal·mol^−1^. Therefore, this unexpected result was further analyzed using both structural and energetic factors. 

**Figure 10:**
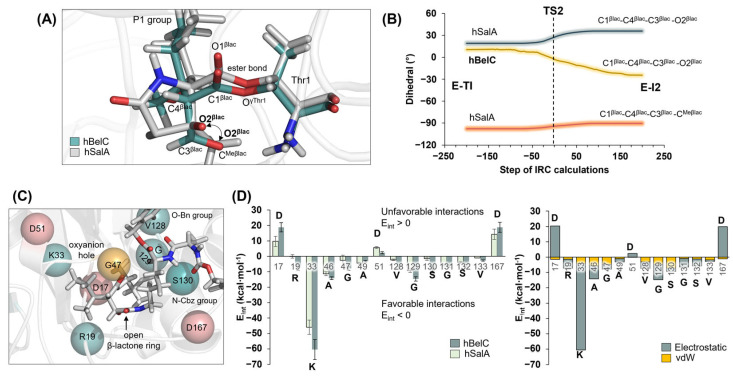
(**A**) Overlay of the optimized structures of E-I2 complexes formed between the active site of β5-subunit and hBelC or hSalA. For clarity, only atoms of warheads and Thr1 are shown. (**B**) The evolution of dihedral angles established between planes defined by positions of C1^βlac^-C4^βlac^-C3^βlac^ -O2^βlac^ and C1^βlac^-C4^βlac^-C3^βlac^-C^Meβlac^ atoms of the β-lactone ring along the C1^βlac^-O2^βlac^ bond cleavage pathway. (**C**) The active site of 20S proteasome in its E-I2 complex with hBelC. Positions of the residues with favorable interactions are shown as cyan spheres while the residues with unfavorable interactions are shown as pink spheres. G47 participating in the formation of the oxyanion hole is shown as an orange sphere. (**D**) Interaction energy (E_int_) established between hBelC or hSalA inhibitors and residues from the vicinity of both warheads in E-I2 adduct together with electrostatic E_elec_ and E_vdW_ contribution to the overall E_int_ values in hBelC. The average values of Eint were obtained based on 1000 structures generated during 100 ps of QM/MM MD simulations at AM1/AMBER level of theory. For clarity, only those residues for which the obtained difference in computed E_int_ for studies inhibitors was higher than 1 kcal·mol^−1^ are provided.

First, the structure of E-I2 intermediates formed between β5-subunit and hBelC and hSalA were compared by the geometrical overlay of the active sites. The result of this structural alignment is shown in Figure 10A. It was observed that although atoms of the P1 group, three carbons of the β-lactone ring, and the oxygen atom of the carbonyl group (O1^βlac^) from both inhibitors occupy almost identical positions in the active site, the orientation adopted by the oxygen (O2^βlac^) of the open ring in the case of hBelC, is meaningfully deviated from the one observed for hSalA. As indicated in Figure 10A, this O2^βlac^ atom of hBelC, besides the translation, experiences additional rotation during C1^βlac^-O2^βlac^ bond cleavage that resulted in changing its position towards the one originally occupied by a methyl group (–CH_3_) in the lactam ring in hSalA. This atom rearrangement was confirmed by a comparative analysis of the evolution of dihedral angles defined by the positions of C1^βlac^-C4^βlac^-C3^βlac^-O2^βlac^ atoms in hBelC, and C1^βlac^-C4^βlac^-C3^βlac^-O2^βlac^ and C1^βlac^-C4^βlac^-C3^βlac^-C^Meβlac^ atoms in hSalA, respectively, which were collected along the second step of inhibition, that corresponds to C1^βlac^-O2^βlac^ bond cleavage. The evolution of dihedrals shown in Figure 10B indicates that in order to accomplish this chemical step, the C1^βlac^-O2^βlac^ bond scission in hBelC is accompanied by the β-lactone ring puckering inversion. The dramatic change of puckering coordinate from c.a. 11° in E-TI to c.a. −3° in TS2 was observed for the four-member ring of hBelC. Puckering inversion of the β-lactone ring was not observed in the case of hSalA for which values of dihedral defined by C1^βlac^-C4^βlac^-C3^βlac^-O2^βlac^ atoms changes from c.a. 19° in E-TI to 27° in TS2.

Since it is expected that such an evident conformational change as the one observed for hBelC cannot go unnoticed by the environment, the next step of the analysis was to test if the better stabilization of E-I2 complex in the case of this inhibitor can originate in the improvement of its interactions established within the active site, by comparison with hSalA. For this purpose, interaction energy (E_int_), as a sum of electrostatic (E_elec_) and vdW (E_vdW_) interactions, was computed for hSalA and hBelC in their E-I2 adducts. The results of these calculations are presented in Figure 10C,D. For clarity, only those residues for which the obtained difference in computed E_int_ for studied inhibitors was higher than 1 kcal·mol^−1^ were provided. During the analysis, it was found that despite two residues, i.e., Asp17, and Asp167 that increase meaningfully their unfavorable interactions with hBelC with respect to hSalA (from 9.7 ± 3.3 to 18.8 ± 3.0, and from 14.2 ± 3.4 to 18.8 ± 3.2 kcal·mol^−1^, respectively), the conformational change in hBelC resulted in gaining much more favorable contacts. Thus, a meaningful rise in the favorable interactions was observed (higher than 5 kcal·mol^−1^ by comparison to E_int_ values computed in E-I2 for hSalA) for hBelC interacting with Arg19, Lys33, Val128, Gly129, and Ser130 residues that can be considered as a key for stabilizing newly formed E-I2 complex. In all cases, the dominant contribution of E_elec_ to the overall value of E_int_ is observed. Importantly, it was found that the interaction with Gly47 from the oxyanion hole was also affected by the conformational change as reflected by the higher value of E_int_ computed for hBelC in comparison to hSalA, i.e., −7.8 ± 1.3 versus −2.6 ± 3.3 kcal·mol^−1^, respectively. 

Regardless the E-I2 complex formation was found to be thermodynamically favored for hBelC by comparison with the same intermediate obtained for hSalA inhibitor, the formation of its structure did not ensure overall product stability. Thus, as commented before, the neutralization of the active site by double proton transfer was required to obtain the desired irreversible product of inhibition. As delivered from calculations, to reach the final product complex (E-PC) the system must cross over an additional not negligible free energy barrier of 19.1 kcal·mol^−1^. However, the newly created E-PC was found to be much more stable in Gibbs free energy, 15.6 kcal·mol^−1^ lower than the non-covalent E•hBelC complex and 7.1 kcal·mol^−1^ lower in comparison to the final product of inhibition formed with hSalA (E-hSalA). This higher stability of the final product in the case of hBelC can be explained using the same arguments as discovered in the analysis carried out for the E-I2 complex. Therefore, and contrary to hSalA which was proven to reversibly inhibit the β5-subunit of the 20S proteasome, the inhibition reaction with hBelC seems to present an irreversible character, because the reversible process that would lead to non-covalent adduct from E-PC requires to surmount the barrier of 40.5 kcal·mol^−1^, what it is rather unfeasible. 

**Figure 11:**
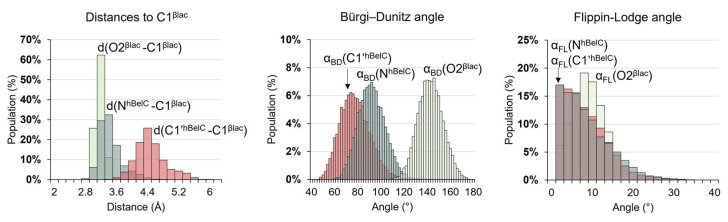
Distribution of distances established between O2^βlac^, N^hBelC^, and C1′^hBelC^ to C1^βlac^ together with the distribution of Bürgi–Dunitz (α_BD_) and Flippin-Lodge (α_FL_) angles obtained for 10,000 snapshots generated during 100 ns MD simulations on E-PC complex.

**Figure 12:**
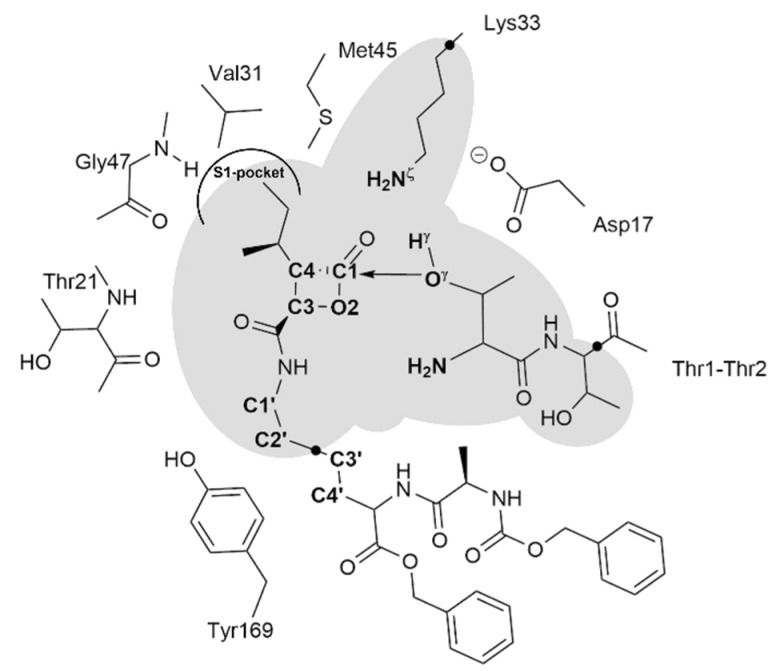
Schematic representation of the active site of β5 subunit of *human* 20S proteasome with hBelC. The grey area includes atoms described at the QM level of theory. The position of link atoms is depicted as three black dots.

The remaining alternative pathway to recover the activity of the enzyme, once the E-PC is formed is through the hydrolysis of the ester bond formed between the Thr1 and C1^βlac^ atom of the lactam ring. The process of hydrolysis would follow a standard reaction mechanism pathway in which in the first step water molecule would attack the C1^βlac^ atom. This step can be achieved within a reasonable energetic cost only if the initial position of the water molecule is geometrically favorable. In other words, the nucleophilic attack trajectory for this molecule should not be disturbed by the presence of another residue from the enzyme or substituents of the inhibitor. In fact, we have previously provided quantitative proof that in the case of hSalA water molecules cannot access the C1^βlac^ atom due to the presence of the hydroxyl group in opened β-lactone ring that resulted in very high free energy barriers for this process. Moreover, it was also demonstrated that the attack on the planar electrophilic center can be achieved only from one side that is opposite to the localization of the highly hydrophobic pocket occupied by the P1 group. [76] Very useful in this analysis appears to be the evaluation of the behavior of the distance established between O2^βlac^ and C1^βlac^ atoms as well as α_BD_ and α_FL_ angles in the E-PC complex. Therefore, in the case of hBelC, results of 100 ns MD simulations obtained for the E-PC model were analyzed in this respect. The distribution of chosen distances together with α_BD_ and α_FL_ angles explored during MD simulations is provided in Figure 11. In this analysis, not only the relative position of O2^βlac^ to C1^βlac^ was examined but also of N^hBelC^ and C1′^hBelC^ atoms of an aminocarbonyl side chain. The atom positions in the structure of hBelC are given in Figure 12. 

As commented before, the optimal position for the nucleophilic attack would be defined by a reasonable short distance to the electrophilic center complemented with a suitable location of the nucleophile in the three-dimensional space, which can be judged based on α_BD_ and α_FL_ values. If any of chosen herein atoms for analysis fulfill these requirements it can be assumed that it creates a steric hindrance and prevents water access to the target of its attack. Since this role was proven to be played by O2^βlac^ in previous work, our analysis of hBelC inhibitor started from this atom. Despite, the distance between O2^βlac^ and C1^βlac^ being short (3.3 ± 0.1 Å) in the E-PC complex, as expected, the α_BD_ is far from the ideal value (105°) and it turned out to be much more obtuse (140 ± 1°) than the same angle of 104° determined for inhibition product created with hSalA along the full relaxation of the system along MD simulations. [76] This change must be dictated by the rotation experienced by O2^βlac^ along the second step of the reaction, which, as discussed before, induces higher stabilization of the E-I2 but at the same time, it exposes access to the C1^βlac^ carbon. Nevertheless, it was observed that due to the characteristic of the hBelC structure, a nitrogen atom, N^hBelC^ of an aminocarbonyl side chain can take over the role of O2^βlac^ and prevent hydrolysis. Considering the preserved short N^hBelC^-C1^βlac^ distance of 3.3 ± 0.1 Å and the very promising α_BD_ and α_FL_ values of 91 ± 1° and 1 ± 1°, it can be concluded that the hydrolysis of E-PC is rather unfeasible. Therefore, the results of this analysis agree with the previous proposal suggesting that hBelC follows a unique mechanism preventing cleavage of its covalent adduct formed with the active site [66].

Finally, the short distance of 2.7 Å established between O2^βlac^ and oxygen of Arg19 (O^Arg19^) of the protein backbone that was observed in the X-ray structure of the E-hBelC [66], was also analyzed and was found to be long (3.68 ± 0.25 Å) in the structure obtained after computational exploration of the last process of the inactivation step. However, as it was observed during longer MD simulations the contact between the hydroxyl group of open lactone ring and Arg17 is oscillating between 2.7 and 7.1 Å (as shown in Figure S1 of Supplementary Materials) indicating that the H-bond interaction established between these atoms in the case of the *human* 20S proteasome and in the given condition of temperature is very labile.

## 3. Computational Methods

### 3.1. System Setup 

The molecular model for the inhibition studies of *human* 20S proteasome with hBelC was prepared using the crystal structure of the CP (PDB ID: 5LF1) [83] with bound SalA molecule. This proteasome-SalA complex was prepared and used in our previous studies. [73] For the purpose of this work, SalA was substituted by the structure of hBelC in its final inhibition product as found in the crystal structure of 20S proteasome from *Saccharomyces cerevisiae* (PDB ID: 3E47) [66] Missing force field parameters for the hBelC covalent adduct was obtained using the Antechamber software [84] (see Table S3 of Supplementary Materials). The pKa shift of all titratable residues was predicted with PropKa software ver. 3.1 [85,86], providing the same results and consequently the same protonation states at pH 7.0 as described in our previous work. [72] Missing hydrogen atoms were added to the enzyme together with 43 positively charged sodium counterions that were put in the most electrostatically favorable positions in order to neutralize the total negative charge of the system. Hydrogen and counterions were added using the tLEAP [87] module of the AmberTools package. Subsequently, the system was soaked within an orthorhombic box of TIP3P [88] water molecules, with a size of 17.1 × 16.7 × 20.0 nm^3^ providing a model consisting of 542,592 atoms. To describe the protein and water molecules the AMBER [89] and TIP3P force fields, respectively, were employed and the NAMD [90] software was used as an MD engine. The equilibration protocol for MD simulations involved a preliminary minimization and gradual heating of the system to 310 K with 0.001 K temperature increments, followed by 100 ps of non-biased NPT equilibration and 100 ps of non-biased NVT equilibration. The final model was used as starting structure for long NVT MD simulations. A cut-off for non-bonding interactions was set between 14.5 to 16 Å using a smooth switching function. Although improved algorithms to compute non-electrostatic interactions have been recently developed [91,92] in this work interactions between the QM and MM moieties were treated by means of standard Lennard-Jones potential [93]. During 100 ns of MD simulations, in order to significantly improve time and reduce the cost of calculations, the positions of all residues beyond 40 Å from the inhibitor were fixed. 40 Å is slightly higher than the double size of radius chosen as the external limit for the cut-off function. The temperature during the simulations was controlled using the Langevin thermostat, [94] and the pressure for the NPT equilibration with the Nosé-Hoover Langevin piston [95] pressure control. 

All simulations were analyzed according to the evolution of the RMSD of the protein backbone as well as of heavy atoms of hBelC and key distances. The system was considered equilibrated after 50 ns of MD simulations (see Figure 4B) and Figure S2 of Supplementary Materials). 

### 3.2. QM/MM Calculations

A representative structure of the product complex E-hBelC from the MD simulation was selected as the starting point for exploring the inhibition mechanism. The size of the system was reduced, cutting the box of waters to a water sphere of 60 Å centered on residue Thr1 of the β5 active site, leading to a system with 149,520 atoms. The AMBER and TIP3P force fields, as implemented in the fDynamo library [96,97], were used for describing the protein, counterions, and water solvent. The cut-off scheme for non-bonding interactions was the same as the one used in the classical MD simulations protocol. Additionally, the positions of the atoms of residues beyond 20 Å from the inhibitor were fixed. The QM part of the model was selected including only the essential 72 atoms, as shown in Figure 12. This includes part of the inhibitor, where the warhead is contained, full N-termini Thr1 residue, part of residue Thr2, and the backbone of residue Lys33. Three-link atoms [98] were inserted on the QM/MM boundaries, placed in the Cα-Cβ bond of Lys33, the C-Cα bond of Thr2, and C2′-C3′ bond in the aliphatic chain of the inhibitor. The Austin Model, AM1 [99] semiempirical Hamiltonian, and the Minnesota density functional, M06-2X [100] with the standard 6-31+G(d,p) basis set, were employed to treat the QM sub-set of atoms. 

### 3.3. Potential Energy Surfaces (PES)

Adequate distinguished reaction coordinates, ξ, were selected for exploring each chemical step of the inhibition mechanism with hBelC. Each PESs was generated by grid scanning, where the step size for hydrogen transfer was controlled every 0.05 Å, while distances between heavy atoms were changed by 0.1 Å. The resulting PES (shown in Figure S3 of Supplementary Materials) allowed identifying the minimum energy path (MEP). All stationary points observed along the MEP were optimized at a low and high level of theory (AM1/AMBER and M06-2X/AMBER, respectively) employing a micro-macro iteration method using Baker’s algorithm [101] and they were characterized by computing the matrix of the second energy derivatives. All computed TSs at M06-2X/MM (the Cartesian coordinates for QM atoms are provided in Table S4 of Supplementary Materials) were connected to the expected minima by tracing intrinsic reaction coordinate (IRC) paths, to further confirm the explored inhibition mechanism. 

### 3.4. Free Energy Surfaces (FES)

A series of QM/MM MD simulations employing the umbrella sampling (US) method [102] as implemented in fDynamo, were computed using previously generated structures at PES at the AM1/MM level of theory. A force constant of 2500 kJ·mol^−1^·Å^−2^ was employed to constrain the reaction coordinate and 310 K was set as the simulation temperature. An initial equilibration of 5 ps was carried out, followed by 20 ps of production at every window. Finally, the weighted histogram analysis method (WHAM) [103] was used to integrate the obtained results in terms of potentials of mean force (PMF). A density tolerance of 10^−3^ to consider the WHAM calculation converged.

The PMF was obtained as a function of the distinguished reaction coordinate, *W*(ξ). The PMF is related to the normalized probability of finding the system at a particular value of the chosen coordinate, as shown in Equation (2),

(2) $$W\left(\xi \right)=C-kTln\int \rho \left(r^{N}\right)\delta \left(\xi \left(r^{N}\right)-\xi \right)dr^{N-1}$$

Then, the free energy of activation can be expressed as follows:

(3) $${\Delta G}^{‡}\left(\xi \right)=W\left(\xi^{‡}\right)-\left[W\left(\xi^{R}\right)+G_{\xi }\left(\xi^{R}\right)\right]$$

where the superscripts indicate the value of the reaction coordinate at the reactants (R), and at the TS (‡), and *G*_ξ_(ξ_*R*_) is the free energy associated with setting the reaction coordinate to a specific value at the reactant state. Normally this last term makes a small contribution, and the activation free energy is directly estimated from the PMF change between the maximum of the profile and the reactant’s minimum,

(4) $${\Delta G}^{‡}\left(\xi \right)\approx W\left(\xi^{‡}\right)-W\left(\xi^{R}\right)=\Delta W^{‡}\left(\xi \right)$$

The selection of the reaction coordinate is usually trivial when the mechanism can be driven by a single internal coordinate or a simple combination (as the antisymmetric combination of two interatomic distances). However, this is not the case for the reaction steps explored in this work. Instead, it was necessary to obtain a much more computationally demanding 2D-PMF using two coordinates: ξ_1_ and ξ_2_. The 2D-PMF is related to the probability of finding the system at distinct values of these two coordinates,

(5) $$W\left(\xi \right)=C^{\prime }-kTln\int \rho \left(r^{N}\right)\delta \left(\xi_{1}\left(r^{N}\right)-\xi_{1}\right)\delta \left(\xi_{2}\left(r^{N}\right)-\xi_{2}\right)dr^{N-2}$$

To estimate the free energy barriers from this quantity, the one-dimensional PMF changes were recovered and a maximum probability reaction path on the 2D-PMF surface was traced integrating over the perpendicular coordinate.

### 3.5. Spline Corrections (SP)

To improve the quality of obtained results and to reduce the possible errors associated with the semiempirical method used during free energy simulations, high-level corrections were employed using DFT method. Spline corrections were applied through the energy function [104,105,106]:

(6) $$E=E_{LL/MM}+S\left[\Delta E_{LL}^{HL}\left(\xi_{1},\xi_{2}\right)\right]$$

where the final energy is obtained from a correction term computed using the single-point energy difference between the high-level (HL) and the low-level (LL) for the QM sub-set of atoms. As mentioned above, the AM1 method was applied as LL method while as the HL the hybrid M06-2X functional with the standard 6-31+G(d,p) basis set was used. The Gaussian09 [107] program combined with fDynamo was employed for DFT/AMBER calculations. In this work, a total of 1798 and 1568 US windows and the respective single-point calculations at the M06-2X/MM level were computed to produce the final QM/MM free energy profiles. 

## 4. Conclusions

In this work, the key molecular factors that are responsible for the irreversible character of hBelC in the covalent inhibition of β5-subunit of *human* 20S proteasome have been revealed using classical MD and QM/MM MD simulations. The herein studied β-lactone containing inhibitor, which is a close analog to a natural product, BelC, seems to be a good candidate because it reveals promising activity against human pancreoma and colon cancer.

The present study provides insights into the recognition and inactivation steps that must be completed to successfully block the activity of the enzyme according to the covalent inhibition mechanism. It shows that hBelC consists of two key fragments that ensure the recognition step, i.e., the sec-butyl and O-benzyl group in P1 and P2′ positions, respectively. Both substituents bind in hydrophobic cavities that ensure favorable weak nonbonding interactions. Moreover, results suggest that while the presence of sec-butyl is crucial as it is responsible for the positioning of the warhead in the active site, the role of the substituent present at P2′ position is rather secondary.

The study of the inactivation step reveals that hBelC irreversibly inhibits the β5-subunit according to the same molecular mechanism as the one observed on the inhibition with hSalA. [76] The most probable reaction mechanism based on the computed free energy profiles indicates that hBelC blocks the active site of the enzyme according to the same sequence of chemical events as previously described for hSalA. Although all three steps of reaction were found to be identical, the computed free energy profile showed meaningful differences. The activation barrier for the rate-limiting step that corresponds to the nucleophilic attack of Thr1 on C1^βlac^ carbon is higher than the one reported previously for hSalA. Computational results revealed that the observed rise in the barrier is due to the weaker electrophilic center on C1^βlac^ offered by hBelC, as well as the longer distance established between nucleophile and electrophile in Michaelis complex, E•hBelC than in E•hSalA. Additionally, stronger stabilization of the final product complex ensures the irreversible character of hBelC which is, in fact, opposite to the reversible behavior of hSalA. Based on our analysis it was concluded that the higher stability of the final covalent complex E-hBelC is related to the conformational change of the β-lactone ring where the flexible oxygen experiences additional rotation during reaction progress, which causes the appearance of more favorable interactions with the residues of the active site. 

The existence of an alternative hydrolysis pathway allowing the recovery of the β5-subunit activity was also excluded. The results of our studies have provided quantitative evidence that hydrolysis of the final product of inhibition with hBelC is unfeasible due to the presence of the aminocarbonyl side chain which most probably blocks the water molecule access to the electrophilic center by occupying the most optimal position for nucleophilic attack in the active site.

We strongly believe that the knowledge gained in this study for the inhibition mechanism of the *human* 20S proteasome with hBelC explored at the atomistic level may be useful in the future. This can be especially relevant in redesigning structures of the already known candidates or designing new ones with additional properties such as for instance polypharmacological anticancer drugs, where hBelC could serve as a template for the dual inhibition of the proteasome and fatty acid. Finally, the results obtained in this work can additionally serve as a reference in a future study on the 20S immunoproteasome providing evidence confirming or contradicting the hypothesis that small sequence changes observed in the primed site of β5i subunit are responsible for increased hBelC affinity towards its binding pocket.

## Data Availability

Data is contained within the article or Supplementary Materials.

## Supplementary Materials

The following supporting information can be downloaded at: https://www.mdpi.com/article/10.3390/ph15050531/s1. Table S1: Key distances for stationary structures optimized and characterized at M06-2X/AMBER level of theory with 6-31+(d,p) basis set, along 20S proteasome inhibition pathway with hBelC. Table S2: ESP charges on key atoms computed for stationary structures optimized and characterized at M06-2X/AMBER level of theory with 6-31+(d,p) basis set. Figure S1: Evolution of the distance established between O2^βlac^ and O^Arg19^ during 100 ns MD simulation in E-PC complex. Table S3: Force field parameters for the hBelC covalent adduct formed with Thr1 of β5 subunit of 20S proteasome. Figure S2: Time-dependent evolution of the total energy, temperature, RMSD of the protein backbone, RMSD of the atoms of hBelC and evolution of key distances during 100 ns of classical MD simulations in E-PC complex. Figure S3: Free energy surfaces computed at AM1/AMBER level for a full three-step inhibition process.; Table S4: Cartesian coordinates of QM atoms of optimized and characterized transition state structures at M06-2X/AMBER level of theory with 6-31+(d,p) basis set, along 20S proteasome inhibition pathway with hBelC.
