# Supplementary material for: Covalent Inhibition of the Human 20S Proteasome with Homobelactosin C Inquired by QM/MM Studies
Source: Pharmaceuticals (Basel). 2022 Apr 25;15(5):531. doi: 10.3390/ph15050531 (PMC9143130; doi:10.3390/ph15050531)
Supplement: Supplementary file 1 [file pharmaceuticals-15-00531-s001.zip › pharmaceuticals-1684355-supplementary.pdf]

Covalent inhibition of the human 20S proteasome with homobelactosin C inquired by QM/MM studies.

Natalia Serrano-Aparicio, Silvia Ferrer, and Katarzyna Świderek*

BioComp Group, Department of Physical and Analytical Chemistry, Universitat Jaume I, 12071 Castellón, Spain.

*Correspondence: Katarzyna Świderek, e-mail address: swiderek@uji.es

Table S1. Key distances for stationary structures optimized and characterized at M06-2X/AMBER level of theory with 6-31+(d,p) basis set, along 20S proteasome inhibition pathway with hBelC.

Distance	E•hBelC	TS1	E-TI	TS2	E-I2	TS3	E-PC
O^{γ} -H^{γ}	1.01	1.37	1.74	1.79	2.25	2.82	3.41
O^{γ} -$C1^{\beta lac}$	2.46	1.80	1.49	1.47	1.33	1.33	1.32
N^{ζ} -H^{γ}	1.64	1.17	1.05	1.05	1.05	1.17	4.09
$C1^{\beta lac}$-$O2^{\beta lac}$	1.33	1.40	1.49	1.53	2.90	2.89	2.98
H^{Thr1}-$O2^{\beta lac}$	3.00	2.76	1.88	1.80	2.33	1.70	0.97
N^{Thr1}-H^{γ}	3.34	3.40	3.60	3.62	3.29	1.46	1.02

Table S2. ESP charges on key atoms computed for stationary structures optimized and characterized at M06-2X/AMBER level of theory with 6-31+(d,p) basis set using ChelpG method, along 20S proteasome inhibition pathway with hBelC.

Atoms	E•hBelC	TS1	E-TI	TS2	E-I2	TS3	PC
O^{γ} (Thr1)	-0.258	-0.302	-0.372	-0.398	-0.432	-0.512	-0.601
H^{γ} (Thr1)	0.053	0.050	0.153	0.124	0.572	0.439	0.266
N^{ζ} (Lys33)	-0.431	-0.229	-0.182	-0.151	-0.469	-0.636	-0.728
N^{Thr1}	-0.895	-0.942	-0.957	-0.936	-0.899	-0.885	-0.423
H^{Thr1}	0.320	0.314	0.307	0.299	0.366	0.534	0.593
$C1^{\beta lac}$	0.512	0.501	0.576	0.723	0.921	1.010	1.227
$O1^{\beta lac}$	-0.658	-0.791	-0.898	-0.890	-0.754	-0.790	-0.814
$O2^{\beta lac}$	-0.158	-0.206	-0.241	-0.328	-1.152	-1.108	-0.887
$C3^{\beta lac}$	-0.135	-0.035	-0.210	-0.156	-0.498	-0.464	-0.926
$C4^{\beta lac}$	0.040	-0.082	0.001	-0.013	0.481	0.663	0.583

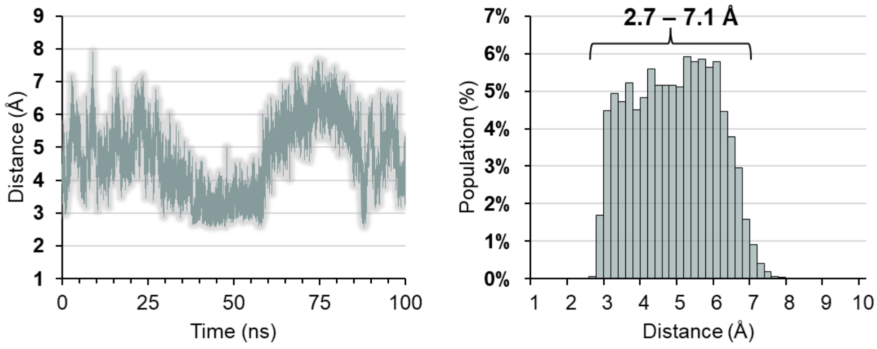

Figure S1. Evolution of the distance established between $O2^{\beta lac}$ and O^{Arg19} for 10 000 snapshots extracted from 100ns MD simulation in E-PC complex (on the left), together with the histogram presenting population size for a range of distances found during simulations.

Table S3. Force field parameters for the hBelC covalent adduct formed with Thr1 of β5 subunit of 20S proteasome (E-hBelC) generated based on GAFF as available in Antechamber software together with the partial charges computed at AM1 level of theory.

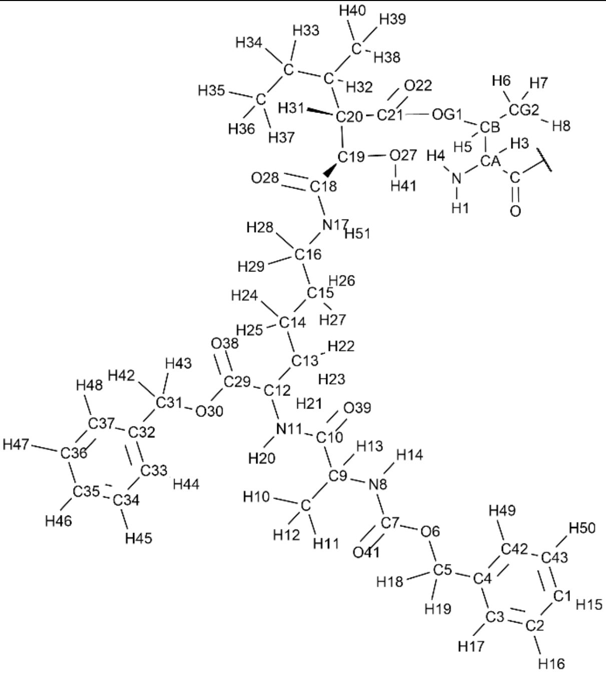

Atom name	Atom type	Charge	Atom name	Atom type	Charge
N	NT	-0.906615	O30	OS	-0.422814
H1	H	0.371376	C31	CT	0.179737
H2	H	0.371376	C32	CA	-0.104279
CA	CT	0.053511	C33	CA	-0.106478
CB	CT	0.156132	C34	CA	-0.137972
CG2	CT	-0.127074	C35	CA	-0.123975
H6	HC	0.053711	C36	CA	-0.137972
H7	HC	0.053711	C37	CA	-0.106478
H8	HC	0.053711	H48	HA	0.154031
OG1	OS	-0.461806	H47	HA	0.131527
C21	C	0.642231	H46	HA	0.128026
C20	CT	-0.139671	H45	HA	0.131527
C19	CT	0.083117	H44	HA	0.154031
C18	C	0.619226	H42	H1	0.066714
N17	N	-0.553787	H43	H1	0.066714
C16	CT	0.09702	O38	O	-0.573883
C15	CT	-0.118376	H21	H1	0.115724
C14	CT	-0.077384	H22	HC	0.065213
C13	CT	-0.09638	H23	HC	0.065213
C12	CT	0.068714	H24	HC	0.04921
N11	N	-0.563785	H25	HC	0.04921
C10	C	0.628228	H26	HC	0.054211
C9	CT	0.051711	H27	HC	0.054211
N8	N	-0.566784	H28	H1	0.061212
C7	C	0.740251	H29	H1	0.061212
O6	OS	-0.399818	H51	H	0.332568
C5	CT	0.176736	O28	O	-0.610975
C4	CA	-0.102279	O27	OH	-0.582681
C3	CA	-0.109978	H41	HO	0.419086
C2	CA	-0.129973	H30	H1	0.116724
C1	CA	-0.120975	C23	CT	-0.054689
C43	CA	-0.129973	C24	CT	-0.089382
C42	CA	-0.109978	C25	CT	-0.092081
H49	HA	0.142529	H35	HC	0.035707
H50	HA	0.135528	H36	HC	0.035707
H15	HA	0.134027	H37	HC	0.035707
H16	HA	0.135528	H33	HC	0.04871
H17	HA	0.142529	H34	HC	0.04871

H18	H1	0.04921	C26	CT	-0.09708
H19	H1	0.04921	H38	HC	0.045376
O41	O	-0.551887	H39	HC	0.045376
H14	H	0.320565	H40	HC	0.045376
C40	CT	-0.113077	H32	HC	0.065713
H10	HC	0.062046	H31	HC	0.108722
H11	HC	0.062046	O22	O	-0.544889
H12	HC	0.062046	H5	H1	0.103721
H13	H1	0.093719	H3	H1	0.086718
O39	O	-0.603977	C	C	0.531008
H20	H	0.353572	O	O	-0.514995
C29	C	0.628228			

Parameters:

**MASS**				**BOND**			
NT	14.010	0.530	same as n3	NT-H	392.40	1.019	same as hn-n3
H	1.008	0.161	same as hn	NT-CT	325.90	1.465	same as c3-n3
CT	12.010	0.878	same as c3	CT-CT	300.90	1.538	same as c3-c3
HC	1.008	0.135	same as hc	CT-H1	330.60	1.097	same as c3-hc
OS	16.000	0.465	same as os	CT-C	313.00	1.524	same as c -c3
C	12.010	0.616	same as c	CT-OS	308.60	1.432	same as c3-os
N	14.010	0.530	same as n	CT-HC	330.60	1.097	same as c3-hc
CA	12.010	0.360	same as c2	OS-C	390.80	1.358	same as c -os
HA	1.008	0.135	same as hc	C -O	637.70	1.218	same as c -o
H1	1.008	0.135	same as hc	CT-OH	316.70	1.423	same as c3-oh
O	16.000	0.434	same as o	C -N	427.60	1.379	same as c -n
OH	16.000	0.465	same as oh	N -CT	328.70	1.462	same as c3-n
HO	1.008	0.135	same as ho	N -H	403.20	1.013	same as hn-n
				CT-CA	326.80	1.510	same as c2-c3
				CA-CA	461.10	1.398	same as ca-ca
				CA-HA	344.30	1.087	same as c2-hc
				OH-HO	371.40	0.973	same as ho-oh

**ANGLE**				**ANGLE**			
NT-CT-CT	66.020	111.040	same as c3-c3-n3	CT-OH-HO	47.380	107.260	same as c3-oh-ho
NT-CT-H1	49.550	109.800	same as hc-c3-n3	C -CT-OH	68.260	108.790	same as c -c3-oh
NT-CT-C	66.320	111.140	same as c -c3-n3	C -CT-H1	46.930	108.770	same as c -c3-hc
H -NT-H	41.400	106.400	same as hn-n3-hn	C -N -CT	63.390	120.690	same as c -n -c3
H -NT-CT	47.420	109.290	same as c3-n3-hn	C -N -H	48.330	117.550	same as c -n -hn
CT-CT-CT	62.860	111.510	same as c3-c3-c3	N -C -O	74.220	123.050	same as n -c -o
CT-CT-OS	68.000	107.970	same as c3-c3-os	N -CT-CT	65.910	111.610	same as c3-c3-n
CT-CT-H1	46.340	109.800	same as c3-c3-hc	N -CT-H1	49.690	109.500	same as hc-c3-n
CT-C -O	67.400	123.200	same as c3-c -o	CT-N -H	45.800	117.680	same as c3-n -hn
CT-CT-C	63.270	111.040	same as c -c3-c3	N -CT-C	67.000	109.060	same as c -c3-n
CT-CT-HC	46.340	109.800	same as c3-c3-hc	N -C -OS	75.320	109.220	same as n -c -os
CT-OS-C	63.280	115.980	same as c -os-c3	OS-CT-CA	68.540	108.560	same as c2-c3-os
HC-CT-HC	39.400	107.580	same as hc-c3-hc	CT-CA-CA	64.060	123.630	same as c2-c2-c3
OS-CT-H1	51.050	108.700	same as hc-c3-os	CA-CT-H1	46.990	110.360	same as c2-c3-hc
OS-C -CT	68.890	110.720	same as c3-c -os	CA-CA-CA	66.620	120.020	same as ca-ca-ca
OS-C -O	75.320	123.250	same as o -c -os	CA-CA-HA	50.010	119.700	same as c2-c2-hc
C -CT-HC	46.930	108.770	same as c -c3-hc	H1-CT-H1	39.400	107.580	same as hc-c3-hc
CT-CT-OH	67.470	110.190	same as c3-c3-oh	OH-CT-H1	51.110	109.500	same as hc-c3-oh
CT-C -N	66.790	115.180	same as c3-c -n				

**DIHEDRALS**					
NT-CT-CT-CT	1	0.156	0.000	3.000	same as X -c3-c3-X
NT-CT-CT-OS	1	0.156	0.000	3.000	same as X -c3-c3-X
NT-CT-CT-H1	1	0.156	0.000	3.000	same as X -c3-c3-X
NT-CT-C -O	1	0.000	180.000	2.000	same as X -c -c3-X
H -NT-CT-CT	1	0.300	0.000	3.000	same as X -c3-n3-X
H -NT-CT-H1	1	0.300	0.000	3.000	same as X -c3-n3-X
H -NT-CT-C	1	0.300	0.000	3.000	same as X -c3-n3-X
CT-CT-CT-HC	1	0.160	0.000	3.000	same as hc-c3-c3-c3
CT-CT-OS-C	1	0.383	0.000	-3.000	same as c3-c3-os-c
CT-CT-OS-C	1	0.800	180.000	1.000	same as c3-c3-os-c
CT-CT-C -O	1	0.000	180.000	2.000	same as X -c -c3-X
CT-OS-C -CT	1	2.700	180.000	-2.000	same as c3-c -os-c3
CT-OS-C -CT	1	0.000	0.000	-1.000	same as c3-c -os-c3
CT-OS-C -CT	1	1.150	0.000	3.000	same as c3-c -os-c3
CT-OS-C -O	1	2.700	180.000	-2.000	same as o -c -os-c3
CT-OS-C -O	1	1.400	180.000	1.000	same as o -c -os-c3
CT-CT-CT-H1	1	0.160	0.000	3.000	same as hc-c3-c3-c3
CT-CT-CT-C	1	0.156	0.000	3.000	same as X -c3-c3-X
HC-CT-CT-OS	1	0.000	0.000	-3.000	same as hc-c3-c3-os
HC-CT-CT-OS	1	0.250	0.000	1.000	same as hc-c3-c3-os
HC-CT-CT-H1	1	0.150	0.000	3.000	same as hc-c3-c3-hc
OS-CT-CT-H1	1	0.000	0.000	-3.000	same as hc-c3-c3-os
OS-CT-CT-H1	1	0.250	0.000	1.000	same as hc-c3-c3-os
OS-CT-CT-C	1	0.156	0.000	3.000	same as X -c3-c3-X
OS-C -CT-CT	1	0.000	180.000	2.000	same as X -c -c3-X
OS-C -CT-HC	1	0.000	180.000	2.000	same as X -c -c3-X

C -OS-CT-H1	1	0.383	0.000	3.000	same as X -c3-os-X
C -CT-CT-C	1	0.156	0.000	3.000	same as X -c3-c3-X
C -CT-CT-OH	1	0.156	0.000	3.000	same as X -c3-c3-X
C -CT-CT-H1	1	0.156	0.000	3.000	same as X -c3-c3-X
C -CT-CT-HC	1	0.156	0.000	3.000	same as X -c3-c3-X
CT-CT-C -N	1	0.100	0.000	-4.000	same as c3-c3-c -n
CT-CT-C -N	1	0.070	0.000	2.000	same as c3-c3-c -n
CT-CT-OH-HO	1	0.160	0.000	-3.000	same as ho-oh-c3-c3
CT-CT-OH-HO	1	0.250	0.000	1.000	same as ho-oh-c3-c3
CT-CT-CT-CT	1	0.180	0.000	-3.000	same as c3-c3-c3-c3
CT-CT-CT-CT	1	0.250	180.000	-2.000	same as c3-c3-c3-c3
CT-CT-CT-CT	1	0.200	180.000	1.000	same as c3-c3-c3-c3
CT-C -N -CT	1	0.000	0.000	-2.000	same as c3-c -n -c3
CT-C -N -CT	1	1.500	180.000	1.000	same as c3-c -n -c3
CT-C -N -H	1	2.500	180.000	2.000	same as X -c -n -X
C -CT-OH-HO	1	0.167	0.000	3.000	same as X -c3-oh-X
C -N -CT-CT	1	0.500	180.000	-4.000	same as c3-c3-n -c
C -N -CT-CT	1	0.150	180.000	-3.000	same as c3-c3-n -c
C -N -CT-CT	1	0.000	0.000	-2.000	same as c3-c3-n -c
C -N -CT-CT	1	0.530	0.000	1.000	same as c3-c3-n -c
C -N -CT-H1	1	0.000	0.000	2.000	same as X -c3-n -X
N -C -CT-OH	1	0.000	180.000	2.000	same as X -c -c3-X
N -C -CT-H1	1	0.000	180.000	2.000	same as X -c -c3-X
N -CT-CT-CT	1	0.156	0.000	3.000	same as X -c3-c3-X
N -CT-CT-HC	1	0.156	0.000	3.000	same as X -c3-c3-X
CT-N -C -O	1	2.500	180.000	2.000	same as X -c -n -X
CT-CT-N -H	1	0.000	0.000	2.000	same as X -c3-n -X
N -CT-C -OS	1	0.000	180.000	2.000	same as X -c -c3-X
N -CT-C -O	1	0.000	180.000	2.000	same as X -c -c3-X
N -C -CT-N	1	1.700	180.000	-1.000	same as n -c3-c -n
N -C -CT-N	1	2.000	180.000	2.000	same as n -c3-c -n
C -N -CT-C	1	0.850	180.000	-2.000	same as c -n -c3-c
C -N -CT-C	1	0.800	0.000	1.000	same as c -n -c3-c
C -CT-N -H	1	0.000	0.000	2.000	same as X -c3-n -X
CT-N -C -OS	1	2.500	180.000	2.000	same as X -c -n -X
N -C -OS-CT	1	2.700	180.000	2.000	same as X -c -os-X
C -OS-CT-CA	1	0.383	0.000	3.000	same as X -c3-os-X
OS-C -N -H	1	2.500	180.000	2.000	same as X -c -n -X
OS-CT-CA-CA	1	0.000	0.000	2.000	same as X -c2-c3-X
CT-CA-CA-CA	1	6.650	180.000	2.000	same as X -c2-c2-X
CT-CA-CA-HA	1	6.650	180.000	2.000	same as X -c2-c2-X
CA-CA-CA-CA	1	3.625	180.000	2.000	same as X -ca-ca-X
CA-CA-CA-HA	1	6.650	180.000	2.000	same as X -c2-c2-X
CA-CA-CT-H1	1	0.380	180.000	-3.000	same as hc-c3-c2-c2
CA-CA-CT-H1	1	0.000	0.000	-2.000	same as hc-c3-c2-c2
CA-CA-CT-H1	1	1.150	0.000	1.000	same as hc-c3-c2-c2
HA-CA-CA-HA	1	6.650	180.000	2.000	same as X -c2-c2-X
O -C -N -H	1	2.500	180.000	-2.000	same as hn-n -c -o
O -C -N -H	1	2.000	0.000	1.000	same as hn-n -c -o
H -N -CT-H1	1	0.000	0.000	2.000	same as X -c3-n -X
H1-CT-C -O	1	0.800	0.000	-1.000	same as hc-c3-c -o
H1-CT-C -O	1	0.000	0.000	-2.000	same as hc-c3-c -o
H1-CT-C -O	1	0.080	180.000	3.000	same as hc-c3-c -o
OS-C -CT-H1	1	0.000	180.000	2.000	same as X -c -c3-X
HC-CT-CT-HC	1	0.150	0.000	3.000	same as hc-c3-c3-hc
O -C -CT-OH	1	0.000	180.000	2.000	same as X -c -c3-X
OH-CT-CT-CT	1	0.156	0.000	3.000	same as X -c3-c3-X
OH-CT-CT-HC	1	0.000	0.000	-3.000	same as hc-c3-c3-oh
OH-CT-CT-HC	1	0.250	0.000	1.000	same as hc-c3-c3-oh
HO-OH-CT-H1	1	0.167	0.000	3.000	same as X -c3-oh-X
HC-CT-C -O	1	0.800	0.000	-1.000	same as hc-c3-c -o
HC-CT-C -O	1	0.000	0.000	-2.000	same as hc-c3-c -o
HC-CT-C -O	1	0.080	180.000	3.000	same as hc-c3-c -o
H1-CT-CT-H1	1	0.150	0.000	3.000	same as hc-c3-c3-hc

**IMPROPER**				
CT-O -C -OS	1.1	180.0	2.0	Using default value
CT-N -C -O	1.1	180.0	2.0	Using default value
C -CT-N -H	1.1	180.0	2.0	Using default value
N -O -C -OS	1.1	180.0	2.0	Using default value
CA-CA-CA-CT	1.1	180.0	2.0	Using default value
CA-CA-CA-HA	1.1	180.0	2.0	Using default value

**NONBON**			
NT	1.8240	0.1700	same as n3
H	0.6000	0.0157	same as hn
CT	1.9080	0.1094	same as c3
HC	1.4870	0.0157	same as hc
OS	1.6837	0.1700	same as os
C	1.9080	0.0860	same as c
N	1.8240	0.1700	same as n

CA	1.9080	0.0860	same as ca
HA	1.4870	0.0157	same as hc
H1	1.4870	0.0157	same as hc
O	1.6612	0.2100	same as o
OH	1.7210	0.2104	same as oh
HO	0.0000	0.0000	same as ho

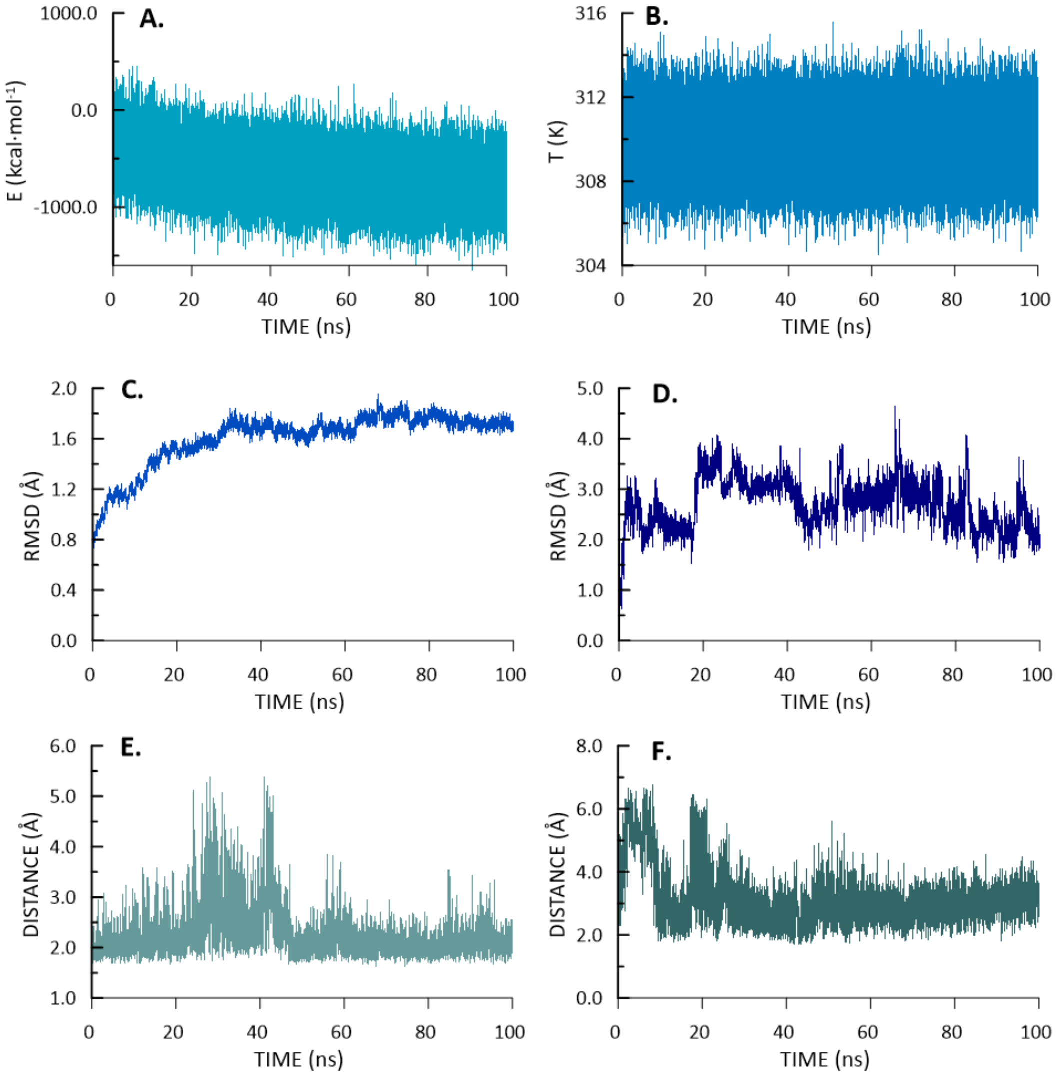

Figure S2. Time-dependent evolution of the total energy (A), temperature (B), the RMSD of the protein backbone (N, Cα, and C atoms; C), the RMSD of the atoms of hBelC, and evolution of key oxyanion hole distances: $O^{\beta lac}$ – H^{Gly47} (E) and $O^{\beta lac}$ – H^{Gly48} (F), along the 100 ns of NVT-MD simulations of 20S proteasome in the E-PC complex form with hBelC covalently bound with Thr1 in the active site of β5 subunit.

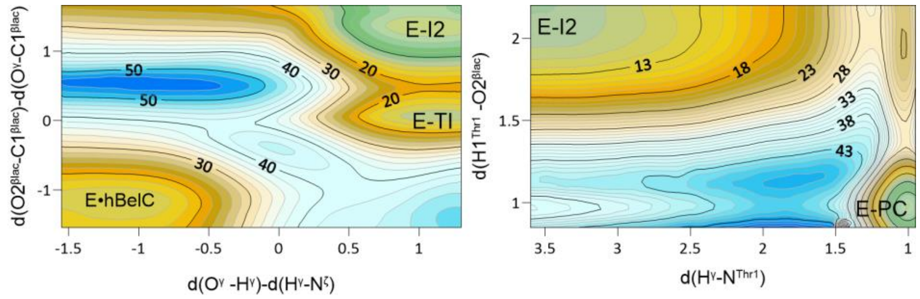

Figure S3. Free energy surfaces computed at AM1/AMBER level for a full three-step inhibition process.

Table S4. Cartesian coordinates of QM atoms of optimized and characterized transition state structures at M06-2X/AMBER level of theory with 6-31+(d,p) basis set, along 20S proteasome inhibition pathway with hBelC.

	TS1			TS2			TS3		
	X	Y	Z	X	Y	Z	X	Y	Z
C	-23.14150	-15.97282	-13.07468	-23.35331	-16.01984	-12.86586	-23.05028	-15.82563	-13.17111
C	-23.40041	-14.48127	-13.34593	-23.41364	-14.51578	-13.29481	-23.22518	-14.33793	-13.34034
C	-23.82223	-14.48094	-11.85429	-23.87237	-14.22071	-11.84728	-23.79317	-13.73986	-11.98180
C	-23.02408	-13.70429	-10.81373	-22.86595	-13.60436	-10.87776	-22.62662	-13.81360	-11.00114
N	-22.96486	-14.37926	-9.62315	-22.81219	-14.24235	-9.67887	-22.75390	-14.74217	-10.05663
C	-21.63079	-14.71214	-9.09833	-21.50826	-14.61034	-9.11689	-21.61884	-15.17161	-9.26619
C	-20.82303	-15.56616	-10.09943	-20.74408	-15.49531	-10.11798	-20.60487	-15.88357	-10.16727
H	-21.40505	-16.43583	-10.40789	-21.38083	-16.31499	-10.46560	-20.96109	-16.88538	-10.42716
H	-20.65618	-14.96981	-11.00364	-20.53359	-14.88592	-11.00325	-20.51718	-15.31966	-11.09791
H	-21.78493	-15.23953	-8.15610	-21.71058	-15.13262	-8.18014	-21.97965	-15.81444	-8.46554
H	-21.09630	-13.78782	-8.87071	-20.93561	-13.71266	-8.87498	-21.16011	-14.29147	-8.80570
H	-23.56797	-15.19936	-9.63428	-23.42789	-15.04769	-9.67417	-23.65265	-15.21887	-10.05613
O	-22.36634	-12.68398	-11.02168	-22.10287	-12.67217	-11.14772	-21.60945	-13.08894	-11.10997
O	-23.26001	-15.80584	-11.69097	-23.87835	-15.58764	-11.49101	-24.90676	-14.33874	-11.52107
H	-24.90294	-14.47499	-11.66713	-24.85380	-13.73423	-11.75760	-23.90413	-12.65556	-12.18256
C	-24.28823	-14.11101	-14.52625	-24.29797	-14.17134	-14.48673	-24.14053	-14.05617	-14.54008
C	-23.73504	-14.86993	-15.74915	-23.71652	-14.91275	-15.70499	-23.47374	-14.58531	-15.82334
C	-24.46060	-14.59533	-17.06481	-24.46462	-14.68769	-17.01787	-24.46545	-14.92944	-16.93106
H	-25.53885	-14.76415	-16.95800	-25.53765	-14.87356	-16.89280	-25.07133	-15.79061	-16.63148
H	-24.09669	-15.27595	-17.84289	-24.09824	-15.37626	-17.78707	-23.95316	-15.19613	-17.86048
H	-24.31615	-13.56894	-17.41801	-24.34689	-13.66628	-17.39294	-25.13954	-14.09165	-17.14246
H	-23.77738	-15.94246	-15.52986	-23.70270	-15.98425	-15.47335	-22.89756	-15.49272	-15.59880
H	-22.66975	-14.62006	-15.86194	-22.66890	-14.60442	-15.82711	-22.74211	-13.84385	-16.17010
C	-24.34540	-12.59952	-14.74428	-24.41495	-12.66385	-14.70808	-24.49520	-12.57558	-14.66139
H	-23.36585	-12.21762	-15.05516	-23.44255	-12.23358	-14.97214	-23.59402	-11.95246	-14.62999
H	-24.63789	-12.08121	-13.82615	-24.77389	-12.16139	-13.80443	-25.16237	-12.26598	-13.85357
H	-25.06894	-12.33588	-15.52247	-25.11736	-12.43264	-15.51592	-25.00486	-12.37652	-15.60976
H	-25.30487	-14.47287	-14.32277	-25.30862	-14.56341	-14.29402	-25.06301	-14.61659	-14.35662
H	-22.41992	-13.99605	-13.45270	-22.39117	-14.17052	-13.48553	-22.22849	-13.91308	-13.51887
O	-22.31066	-16.76156	-13.52962	-22.23946	-16.62651	-12.90254	-21.99661	-16.39586	-12.92108
N	-25.41150	-17.14337	-10.34097	-25.45663	-17.36726	-10.19245	-26.15745	-16.72415	-11.54158
H	-24.54909	-17.49582	-9.92565	-24.91244	-16.53536	-10.36209	-25.47227	-15.94168	-11.44801
H	-26.12316	-17.30334	-9.62826	-24.97045	-17.96502	-9.52998	-26.65989	-16.88751	-10.67498
C	-25.75127	-18.01090	-11.46681	-25.67123	-18.07889	-11.43544	-25.70956	-17.93801	-12.20497
H	-23.74947	-18.02345	-12.17740	-23.61008	-18.18641	-12.09091	-23.55584	-17.94860	-12.01043
C	-24.73839	-17.90996	-12.64266	-24.58068	-18.04412	-12.57484	-24.30732	-17.81992	-12.79394
H	-26.75252	-17.73272	-11.80021	-26.63217	-17.77088	-11.85756	-26.37720	-18.10020	-13.06055
C	-24.86559	-18.99944	-13.70506	-24.76931	-19.10968	-13.64819	-24.14694	-18.81325	-13.92439
H	-25.87710	-19.04564	-14.12313	-25.77318	-19.06456	-14.08669	-24.98792	-18.72162	-14.61336
H	-24.63429	-19.99764	-13.32421	-24.63710	-20.12430	-13.26820	-24.12744	-19.84234	-13.55674

H	-24.15932	-18.75523	-14.50321	-24.03251	-18.92887	-14.43461	-23.24716	-18.60524	-14.48667
O	-24.79013	-16.65230	-13.28665	-24.52046	-16.79285	-13.29958	-24.20597	-16.47816	-13.29993
H	-26.10517	-16.43427	-13.58974	-26.24862	-16.42422	-13.59876	-26.94056	-16.22674	-12.67372
C	-25.76486	-19.48826	-11.11511	-25.76998	-19.55186	-11.10759	-25.69853	-19.26192	-11.42406
O	-24.86732	-19.96831	-10.40658	-24.90424	-20.08556	-10.39857	-24.81048	-19.51940	-10.60055
N	-26.71338	-20.20205	-11.73372	-26.74536	-20.21032	-11.73447	-26.61492	-20.11648	-11.88224
H	-27.37725	-19.71626	-12.35054	-27.37593	-19.69672	-12.36117	-27.33813	-19.74504	-12.51259
C	-26.59156	-21.63368	-11.94450	-26.63631	-21.63102	-11.98490	-26.43782	-21.55631	-11.95969
H	-25.58911	-21.90852	-11.58664	-25.65927	-21.93646	-11.58621	-25.40526	-21.76739	-11.64433
C	-27.61155	-22.36175	-11.05626	-27.70560	-22.37386	-11.17210	-27.36702	-22.29319	-10.99323
H	-28.61227	-22.21289	-11.49243	-28.68686	-22.19602	-11.64235	-28.40717	-22.09516	-11.29557
C	-27.31286	-23.84886	-10.96001	-27.42966	-23.86800	-11.11530	-27.11680	-23.79341	-11.01127
H	-28.05880	-24.34924	-10.33450	-28.20958	-24.38079	-10.54347	-27.78601	-24.29579	-10.30602
H	-27.30204	-24.30964	-11.95160	-27.37501	-24.29459	-12.12046	-27.27583	-24.19983	-12.01323
H	-26.32936	-23.99015	-10.49739	-26.47164	-24.03733	-10.61136	-26.08377	-24.00314	-10.70978
O	-27.59822	-21.87210	-9.72387	-27.73604	-21.93578	-9.82427	-27.16681	-21.87272	-9.65576
H	-27.56119	-20.89982	-9.72836	-27.69402	-20.96257	-9.77688	-27.37230	-20.92017	-9.56920
C	-27.14332	-18.01753	-18.54916	-27.15884	-17.99904	-18.52725	-27.25453	-17.91679	-18.27203
H	-26.07264	-17.93772	-18.76687	-26.08769	-17.91644	-18.74111	-26.17748	-17.80684	-18.44045
H	-27.25433	-19.00396	-18.09845	-27.26877	-18.98505	-18.07495	-27.34937	-18.90515	-17.82437
C	-27.52947	-16.92785	-17.54686	-27.54903	-16.91084	-17.52340	-27.69642	-16.84217	-17.24229
H	-28.61764	-16.82911	-17.44984	-28.63647	-16.79891	-17.44101	-28.78188	-16.68890	-17.24460
H	-27.15741	-15.96089	-17.90652	-27.16114	-15.94595	-17.87075	-27.25663	-15.87661	-17.52715
C	-27.00333	-17.17748	-16.13528	-27.04322	-17.18051	-16.10566	-27.32500	-17.12932	-15.76490
H	-25.90557	-17.16893	-16.10595	-25.94531	-17.17724	-16.06421	-26.26683	-17.40234	-15.68026
H	-27.32559	-18.16918	-15.79358	-27.37838	-18.17112	-15.77519	-27.90719	-17.97739	-15.38786
C	-27.57747	-16.11148	-15.21329	-27.62208	-16.11439	-15.18798	-27.61330	-15.85828	-14.95730
H	-28.66792	-16.09104	-15.31438	-28.71309	-16.11862	-15.25050	-28.56105	-15.43766	-15.31296
H	-27.20036	-15.11823	-15.47461	-27.26633	-15.11475	-15.45121	-26.85013	-15.10399	-15.16111
N	-27.25569	-16.35505	-13.79197	-27.28444	-16.34374	-13.75803	-27.74811	-15.93653	-13.47100
H	-27.63643	-15.59005	-13.22828	-27.64941	-15.57177	-13.18862	-27.99311	-14.97897	-13.18125
H	-27.73196	-17.22387	-13.47732	-27.74915	-17.21775	-13.42457	-28.54444	-16.54336	-13.19925
